# Effect of a New Fermentation Strain Combination on the Fermentation Process and Quality of Highland Barley Yellow Wine

**DOI:** 10.3390/foods13142193

**Published:** 2024-07-11

**Authors:** Xiaodie Chen, Chuan Song, Jian Zhao, Zhuang Xiong, Lianxin Peng, Liang Zou, Bingliang Liu, Qiang Li

**Affiliations:** 1 Key Laboratory of Coarse Cereal Processing, Ministry of Agriculture and Rural Affairs, Sichuan Engineering & Technology Research Center of Coarse Cereal Industrialization, School of Food and Biological Engineering, Chengdu University, Chengdu 610106, China; cxd0512@126.com (X.C.); xiongzhuang2000@126.com (Z.X.); penglianxin@cdu.edu.cn (L.P.); zouliang@cdu.edu.cn (L.Z.); 2 Luzhou Laojiao Co., Ltd., Luzhou 646000, China; songchuan@lzlj.com; 3 National Engineering Research Center of Solid-State Brewing, Luzhou 646000, China; 4 Postdoctoral Research Station of Luzhou Laojiao Company, Luzhou 646000, China; 5 School of Life Sciences, Sichuan University, Chengdu 610041, China; zj804@163.com

**Keywords:** highland barley, fermentation, yellow wine, volatile compounds, free amino acids

## Abstract

Yellow wine fermented from highland barley is an alcoholic beverage with high nutritional value. However, the industrialization of barley yellow wine has been constrained to a certain extent due to the lack of a systematic starter culture. Therefore, the present study aims to simulate barley yellow wine fermentation using a starter culture consisting of *Rhizopus arrhizus*, *Saccharomyces cerevisiae*, *Pichia kudriavzevii*, and *Lacticaseibacillus rhamnosus*. In this study, changes in enzyme activity, fermentation characteristics, volatile substance production, and amino acid content during the fermentation of highland barley yellow wine brewed with different starter cultures were evaluated. The results of this study show that regulating the proportion of mixed starter bacteria can effectively control the various stages of the fermentation process and improve the organoleptic characteristics and quality of yellow wine to varying degrees. Additionally, we found that the addition of probiotics could effectively improve the palatability of yellow wine. To the best of our knowledge, we have validated for the first time the use of the above multispecies starter culture, consisting of *R. arrhizus*, *S. cerevisiae*, *P. kudriavzevii*, and *L. rhamnosus*, in the production of highland barley yellow wine. The obtained findings provided reference data for optimizing highland barley yellow wine fermentation.

## 1. Introduction

Highland barley yellow wine is a traditional alcoholic drink that originated on the Qinghai–Tibet Plateau of China and has a long history [1]. Yellow wine brewing is a complex microbial metabolic process in which the composition and activity of the microbial flora are key factors affecting the quality and flavor of yellow wine [2]. For example, they can produce a variety of vitamins, amino acids [3], polyphenols [4], trace elements [5,6], and other bioactive substances that are beneficial for promoting digestion, improving immunity, and promoting blood circulation [7,8,9]. At the same time, yellow wine can be used as a flavoring for cooking, providing a certain aroma and deodorizing effect to dishes [10]. The fermentation of traditional highland barley yellow wine is predominantly achieved through spontaneous fermentation, where microorganisms existing in the raw materials, equipment, and surroundings are used for the fermentation process. In the raw fermentation material, wheat koji, there is a blend of bacteria, yeast, and mold, along with several enzymes including alpha-amylase, glucoamylase, and protease [11,12]. Wheat koji from different areas contains different dominant fermenting microorganisms, which endow yellow wine with unique flavors [13,14]. However, there are currently few studies on barley yellow wine. Due to the instability of wheat koji and the lack of a systematic starter culture, the production of highland barley yellow wine is hindered, and the standardization of barley yellow wine is limited to some extent.

Yellow wine fermentation is mainly a synergistic process of acidification, saccharification, alcoholization, and esterification, in which *Rhizopus*, yeasts, and lactic acid bacteria play important roles [15,16]. Numerous studies have shown that *Rhizopus* plays a dominant role in the fermentation process [17,18,19,20]. *Rhizopus* can produce a variety of enzymes during the growth process, including amylase, lipase, cellulase, and others [21]. Yeasts are divided into two main groups: *Saccharomyces cerevisiae* and non-*Saccharomyces cerevisiae*. *S. cerevisiae* mainly breaks down sugars into alcohol and carbon dioxide while metabolically producing organic acids, esters, aldehydes, higher alcohols, and many other flavoring substances, which are essential for the formation of special flavors in yellow wine [22]. Common non-*Saccharomyces cerevisiae* include *Pichia*, *Metschnikowia*, and *Kluyveromyces* [23]. Most of the acids in yellow wine are organic acids, and lactic acid is mainly produced by lactic acid bacteria. Common lactic acid bacteria in yellow wine fermentation are *Lactiplantibacillus plantarum*, *Levilactobacillus brevis* and *Pediococcus pentosaceus* [24]. Li et al. [25] used *Saccharomyces cerevisiae*, *Pichia kudriavzevii*, and *Lactiplantibacillus plantarum* for the co-fermentation of cider, resulting in enhanced antioxidant capacity and a higher content of glucuronic acid. To improve the stability of yellow wine fermentation and product quality, many researchers choose to use a variety of specific microorganisms, especially molds and yeasts, for mixed fermentation [26]. Yu et al. [27] used wheat koji produced by the mixed fermentation of *Aspergillus niger* YF2 and *Rhizopus oryzae* YF1 to ferment yellow wine. During the fermentation process, the activities of amylase, acid protease, and cellulase significantly increased. Based on this, the contents of short peptides and free amino acids in the final fermentation product increased by 19.6% and 131.8%, respectively. Shan Liu et al. [28] found that after passing through *R. chinensis* R01, *A. niger* A20, *M. pusillus* M05, and *S. cerevisiae* S10 (RAM-S), yellow wine had higher contents of ethanol and amino acid nitrogen but a lower content of bitter free amino acids. It also exhibited greater flavor characteristics, such as “fruity aroma” and “floral aroma”.

However, when various microorganisms coexist in a fermentation system, their interaction is the key to ensuring the safety of food fermentation [29]. Therefore, it is particularly important to study the effect of the microbial agent mixing ratio on yellow wine fermentation. Through a reasonable mixing ratio, the metabolites between strains can promote each other, thus improving fermentation efficiency. Different strains of microbial agents also produce various metabolites during fermentation. These metabolites have important effects on the aroma, taste, and color of yellow wine. However, studies on the effect of the mixing ratio of microorganisms in starter cultures on the fermentation process of yellow wine are still limited. Hence, the aim of this research was to examine how the use of various starter cultures with different microbial mixing ratios impacts the fermentation process of highland barley yellow wine. The goal is to establish a solid scientific foundation for the production of liquid fermented yellow wine.

In this study, we evaluated the wine-making performance and compatibility of starters consisting of *Rhizopus arrhizus*, *Saccharomyces cerevisiae*, *Pichia kudriavzevii*, and *Lacticaseibacillus rhamnosus* at different ratios through a yellow wine brewing test. The parameters included fermentation efficiency, alcohol yield, and flavor quality. Finally, the feasibility of the optimized microbial agent mixing ratio was validated by multiple experiments. The results of this study showed that an appropriate ratio of microbial agents could significantly improve the stability of the fermentation process and the product quality of highland barley yellow wine. To our knowledge, this study is the first to describe the fermentation performance of a *R. arrhizus*, *S. cerevisiae*, *P. kudriavzevii*, and *L. rhamnosus* hybrid starter culture in yellow wine brewing.

## 2. Materials and Methods

### 2.1. Microbial Strains

*Rhizopus arrhizus* G01, *Saccharomyces cerevisiae* XDN2, *Pichia kudriavzevii* XDB1, and *Lacticaseibacillus rhamnosus* SL02 were isolated from yellow wine koji from around the world. Genetic characterization was previously performed, and their fermentation performance was studied independently at the laboratory scale. They were deposited in the Department of Food and Bioengineering at Chengdu University. *R. arrhizus*, *S. cerevisiae*, and *P. kudriavzevii* were cultured in PDB media (potato dextrose broth), while *L. rhamnosus* was cultured in MRS broth and stored in 20% glycerol at −80 °C.

### 2.2. Fermentation Process of Highland Barley Yellow Wine

A total of 150 g of highland barley and glutinous rice were soaked in distilled water at a ratio of 1:2 (50 g of highland barley and 100 g of glutinous rice) for 12 h. They were then cooked in a 1200 W induction cooker for 35 min until the grains of highland barley and glutinous rice were distinct. The steamed highland barley and glutinous rice were placed on a sterilized operating table to cool to 30–35 °C. Then, the mold spore suspension was inoculated into the barley and glutinous rice and mixed well. Once the mixture had been transferred to a wine tank, 100 mL of sterile water was added for fermentation at 28 °C, lasting for 5 days. The next step involved fermenting the mixture at 15 °C for a total of 20 days. To enhance the performance of yeast and lactic acid bacteria, a sequential inoculation protocol was employed [30,31,32]. After 24 h of fermentation, *S. cerevisiae*, *P. kudriavzevii*, and *L. rhamnosus* were inoculated. Four hybrid fermentation combination methods were implemented: A (*R. arrhizus* G01:*S. cerevisiae* XDN2:*P. kudriavzevii* XDB1:*L. rhamnosus* SL02 = 4:2:1:1), B (*R. arrhizus* G01:*S. cerevisiae* XDN2:*P. kudriavzevii* XDB1:*L. rhamnosus* SL02 = 5:1:1:1), C (*R. arrhizus* G01:*S. cerevisiae* XDN2:*P. kudriavzevii* XDB1:*L. rhamnosus* SL02 = 4:1:1:2), and D (*R. arrhizus* G01:*S. cerevisiae* XDN2:*P. kudriavzevii* XDB1:*L. rhamnosus* SL02 = 4.5:2.5:0.5:0.5). A total of 8 mL of the four strain suspensions were inoculated and distributed according to the above ratios, with the number of each microorganism controlled at 1 × 10^6^ CFU/mL. The total inoculum volume was 5% of the volume of yellow wine. The control used was a commercially available koji yellow wine. The highland barley yellow wine sample was then obtained by filtering through gauze, clarifying, and pasteurizing at 75 °C for 10 min. Three parallel experiments were performed for each combination. Before the experiments, the collected wine samples were cryopreserved at −20 °C.

### 2.3. Determination of Enzyme Activity during Fermentation

#### 2.3.1. Determination of α-Amylase Activity

The determination of α-amylase activity was performed according to Yoo’s iodine colorimetric method [33]. Five milliliters of a 0.5% starch solution was preheated in a 60 °C water bath for 10 min. Then, 0.5 mL of yellow wine liquid was added, and the mixture was allowed to react at 60 °C for 5 min. After that, the reaction was terminated with 5 mL of 0.1 mol/L hydrochloric acid. Next, 0.5 mL of the reaction solution and 5 mL of a dilute iodine solution were mixed to measure the OD620 of the mixture. The reaction solution was a mixture of yellow wine liquid and starch solution after the addition of hydrochloric acid, as described above. A blank was prepared using 0.5 mL of water, and a control sample was prepared using a phosphate buffer instead of wine. At this point, for every milliliter of wine sample, 1 μg of maltose was produced per minute through the catalysis of starch. This was defined as one unit of enzyme activity (µg/min/mL).

#### 2.3.2. Determination of Glucoamylase Activity

The determination of glucoamylase activity was performed according to the method reported by Li [34]. Twenty-five milliliters of a 2% starch solution was mixed with 5 mL of an acetic acid–sodium acetate buffer and preheated in a water bath at 40 °C for 5 min. Then, 1 mL of yellow wine liquid was added for 30 min, and 0.2 mL of 200 g/L NaOH was used to terminate the reaction. One milliliter of the reaction solution was diluted to ten milliliters with water. As mentioned above, this reaction solution is the mixture formed by the addition of NaOH. Then, 0.4 mL of the diluted solution was mixed with 0.6 mL of DNS, boiled for 5 min, and cooled in ice water. Next, 3 mL of water was added to the cooled mixture. The OD540 was measured. At this time, at 40 °C and pH 4.6, 1 mL of the wine sample was hydrolyzed with soluble starch per minute to produce 1 μg of glucose, which was used as one unit of enzyme activity (μg/min/mL).

#### 2.3.3. Determination of Acid Protease Activity

The determination of acid protease activity was performed using Folin’s method. At this time, 1 μg of tyrosine was produced from the catalytic hydrolysis of each milliliter of wine sample at 40 °C, and this was considered one enzyme activity unit (µg/min/mL).

### 2.4. Analysis of the Physicochemical Parameters of Highland Barley Yellow Wine

The reducing sugars, total acids, and amino acid nitrogen of the yellow wine were measured according to the methods of Wei and Zhang et al. [35,36]. The determination of reducing sugars, using dinitrosalicylic acid (DNS), involves a redox reaction with reducing sugars under alkaline conditions. The total acid content was assessed through titration with 0.1 mol/L NaOH, and the result was presented as lactic acid equivalents. The alcohol content was measured using an alcohol meter.

For the sensory analysis of yellow wine, refer to the work of Wei et al. [35]. We selected 20 trained sensory evaluators to evaluate the color, aroma, taste, and style of the five groups of yellow wine. Each evaluator tasted at least 10 different types of yellow wine to ensure that they had a full understanding of the sensory properties of yellow wine. The personal data and privacy of the researchers were anonymized, and corresponding measures were taken to protect them. Before the trial data were released to the public, unanimous consent was obtained from all participants. The evaluation took place in a typical sensory assessment room. A light-tight cup containing 30 mL of yellow wine was used for the analysis, and the samples were randomly selected in no particular order. To avoid fatigue for the group members, the five samples were divided into groups and evaluated, and each group was evaluated after 15 min. The evaluators quantified each sensory description according to the sensory evaluation standards of yellow wine to express their decisions. Finally, the ratings of the evaluators were collected, and statistical analysis was performed.

### 2.5. Measurements of the Electronic Tongue

The taste was measured using the Electronic tongue (Alpha MOS, Toulouse, France). The sensor was composed of seven sensors: AHS-Source, CTS-Saltiness, NMS-Umami, ANS, SCS, PKS, and CPS. Ag/AgCl was chosen as the reference electrode [37]. Eighty milliliters of wine liquid was placed in a special beaker for electronic tongue detection. Samples CK, A, B, C, and D were placed at positions 4, 5, 6, 7, and 8 of the electronic tongue autosampler. Eighty milliliters of distilled water was placed at positions 1, 2, and 3. Before detecting the samples, the sensor was washed for 120 s, and then it detected for 120 s. Each sample was tested six times, and the stable value between 100 and 120 s for the last five times was taken as the test result for analysis.

### 2.6. HS-SPME–GC–MS Detection

#### 2.6.1. HS-SPME Conditions

The characteristics of the solid-phase microextraction head (Supelco, Bellefonte, PA, USA) are as follows: 50 s/30 μm PDMS/DVB/CAR, Stableflex; the extraction head aging temperature was set to 250 °C with an aging time of 300 s. The insertion depth was 20 mm, and the coating outstretched length was 12 mm. The samples were placed in 20 mL headspace vials and preheated at 40 °C. The extraction temperature was set to 60 °C with an extraction time of 2400 s, an insertion depth of 15 mm, and a coating outstretched length of 12 mm. The stirring rate was set to 300 r/min with a stirring time of 600 s. The resolution temperature was set to 270 °C with a resolution time of 300 s, an insertion depth of 20 mm, and a coating outstretched length of 12 mm.

#### 2.6.2. GC–MS Conditions

An Agilent 19091s-433 HP-5MS column (30 m × 0.25 mm × 0.25 μm, Supelco Inc., Bellefonte, PA, USA) was used for the analysis. The column temperature was programmed to increase, starting at 40 °C for 5 min and then increasing to 70 °C at a rate of 2 °C/min. It was then held at 70 °C for 2 min before being increased to 120 °C at a rate of 3 °C/min. The temperature was then increased to 150 °C at a rate of 5 °C/min and finally to 230 °C at a rate of 10 °C/min. The temperature was held at 230 °C for 2 min. The flow rate was set to 1.4 mL/min. The detector used was an MSD detector, and the carrier gas used was helium (99.999% purity) at a flow rate of 40 mL/min with a shunt ratio of 5:1. The sweep flow was 3 mL/min. The inlet temperature was set to 270 °C. The ion source used was an EI source with an electron energy of 70 eV. The ion source temperature was set to 230 °C, the quadrupole temperature to 150 °C, and the transmission line temperature to 280 °C. The acquisition mode used was scanning mode (Scan) with a scanning mass range of 50–550.

The identification of volatile compounds (presumptive) was conducted with a comparison to the NIST mass spectral library, retrieving individual peaks in the total ion chromatogram and thereby identifying a wide range of volatile compounds. The method used for quantitative analysis was peak area normalization, whereby the relative mass fraction of each compound is expressed as a percentage of the peak area of that component to the sum of the peak areas of all identified components.

### 2.7. Amino Acid Determination

#### 2.7.1. Sample Pretreatment with Hydrolyzed Amino Acids (Minimum Protein Content Requirement: 1 mg/mL)

Five milliliters of the stock solution of the liquid sample (appropriately increased or decreased according to the protein content) was added to the hydrolysis tube. Ten milliliters of analytical grade hydrochloric acid (approximately 6 M) was added at a 1:1 ratio. The tube was then bubbled with nitrogen gas for 30 s and sealed. Next, the tube was placed in an oil bath at 110 °C for 22–24 h. After hydrolysis, the mixture was cooled to room temperature, filtered through a 0.45 μm membrane into a 50 mL volumetric flask, and made up to a constant volume. Then, 2 mL of the final sample was pipetted and placed on a rotary evaporator to deacidify at 45 °C. It was deacidified until dry, leaving a small amount of solid or stain remaining at the bottom of the flask. Then, 2 mL of a sodium citrate buffer was added to fully dissolve it. The mixture was then passed through a 0.45 μm filter. After filtering, the sample was tested using an amino acid analyzer.

#### 2.7.2. Preparation of Standards

The Amino Acid Standard (AAS18) was purchased from Sigma (St. Louis, MO, USA) and was diluted two times with a sodium citrate buffer. The diluted concentration was as follows: aspartic acid 1.25 μM/mL, threonine 1.25 μM/mL, serine 1.25 μM/mL, glutamic acid 1.25 μM/mL, glycine 1.25 μM/mL, alanine 1.25 μM/mL, cysteine 0.625 μM/mL, valine 1.25 μM/mL, methionine 1.25 μM/mL, isoleucine 1.25 μM/mL, leucine 1.25 μM/mL, tyrosine 1.25 μM/mL, phenylalanine 1.25 μM/mL, histidine 1.25 μM/mL, lysine 1.25 μM/mL, arginine 1.25 μM/mL, proline 1.25 μM/mL, and ammonia 1.25 μM/mL.

#### 2.7.3. Buffer

For the amino acid chromatography of protein hydrolysates, three buffers and a regeneration solution (0.04 M sodium hydroxide) were used in the step elution procedure. The first buffer, pH 3.20 and 0.20 M, elutes the amino acids Asp, Thr, Ser, Glu, Pro, Gly, and Ala. On the other hand, Cys was eluted on the gradient of the first and second buffers. The amino acids Val, Met, Ile, Leu, and Nleu were eluted in the second buffer. When amino sugars were present in the sample, the time required to use the second buffer (pH 4.25 and 0.2 M) was extended to elute Tyr and Phe. The third buffer, pH 6.45 and 0.2 M, was used to elute the amino acids His, Lys, Amm, and Arg.

#### 2.7.4. Experimental Conditions of the Amino Acid Analyzer

Detection was performed using a Biochrom 30+ (Biochrom Ltd., Cambridge, UK) machine. The following citrate buffers were used: B1 with a pH of 3.2, B2 with a pH of 4.25, and B5 with a pH of 6.45. The buffer flow rate was 25 mL/h, the reaction flow rate was 10 mL/h, and the separation column was a Na-type cationic resin chromatography column with a length and diameter of 200 mm × 4.6 mm, 8 μm particles, and UV detection wavelengths of 570 nm and 440 nm. The column temperature was 55–65–77 °C, the reaction tank temperature was 138 °C, and the sample injection volume was 20 µL. The detection method involved separating amino acids on a separation column and reacting them with ninhydrin. The next step involved using a spectrophotometer to identify the products and determine the concentration of amino acids (post-column derivatization method).

### 2.8. Statistical Analysis

The analysis of variance (ANOVA) for this study was performed using SPSS 21.0 statistical software, and all tests were repeated three times. A *p*-value of less than 0.05 was considered to indicate statistical significance. The differences in taste expression among the yellow wine samples in the different treatment groups were investigated with principal component analysis (PCA) using Gene Denovo.

## 3. Results and Discussion

The objective of this study was to explore the wine-making performance of a mixed starter culture composed of four isolates, namely *R. arrhizus*, *S. cerevisiae*, *P. kudriavzevii*, and *L. rhamnosus*. In our previous work, we monitored the dynamics of physicochemical parameters during the fermentation of highland barley yellow wine. For example, the total sugar content did not change much at the beginning of fermentation, but it continued to decrease in the later stages. The alcohol content showed a slow increase, followed by a rapid increase, and finally stabilization. The fermentation developed in a similar way to that reported in previous studies [38]. From what we understand, we have validated for the first time the use of the above multispecies starter culture in the production of highland barley yellow wine.

### 3.1. Changes in Enzyme Activities during the Fermentation of Yellow Wine

**Figure 1:**
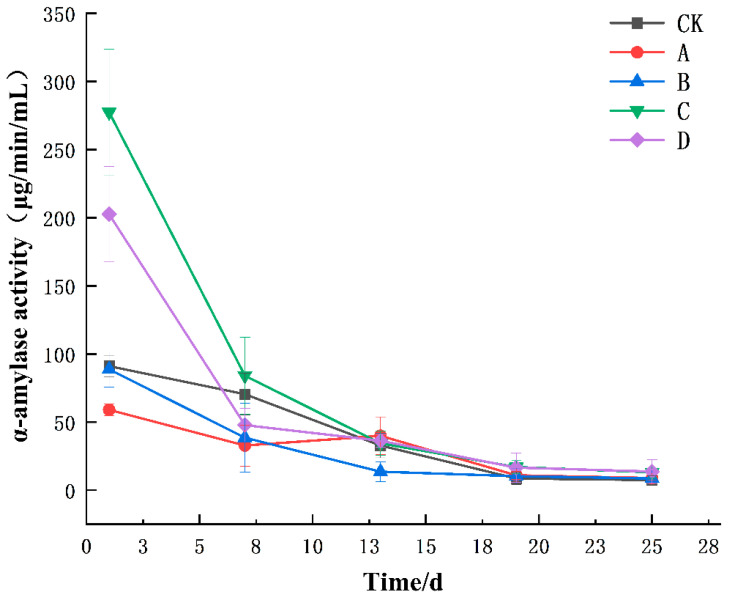
Changes in α-amylase activity during the fermentation process of yellow wine. Groups A, B, C, and D correspond to four starter cultures with different proportions of mixed strains. A (*R. arrhizus* G01:*S. cerevisiae* XDN2:*P. kudriavzevii* XDB1:*L. rhamnosus* SL02 = 4:2:1:1), B (*R. arrhizus* G01:*S. cerevisiae* XDN2:*P. kudriavzevii* XDB1:*L. rhamnosus* SL02 = 5:1:1:1), C (*R. arrhizus* G01:*S. cerevisiae* XDN2:*P. kudriavzevii* XDB1:*L. rhamnosus* SL02 = 4:1:1:2), and D (*R. arrhizus* G01:*S. cerevisiae* XDN2:*P. kudriavzevii* XDB1:*L. rhamnosus* SL02 = 4.5:2.5:0.5:0.5).

α-Amylase can hydrolyze starch into short-chain dextrins of varying lengths and a small amount of low-molecular-weight sugars [39]. As shown in Figure 1, during the entire fermentation cycle of the yellow wine, the α-amylase activity generally decreased. The α-amylase activity of the CK group showed a significant decrease (*p* < 0.05) from day 1 to day 19 during the fermentation of yellow wine, and there was no significant change in the α-amylase activity after day 19. The α-amylase activities of groups C and D were significantly higher than those of the other groups on the first day of fermentation (*p* < 0.05), and the enzyme activities of the five groups of wine samples rapidly declined by the 7th day of fermentation. There were no significant differences in enzyme activities among the five groups on the 19th and 25th days of fermentation. During the initial phases of fermentation, large quantities of mold multiplied, the acidity was low, the temperature was suitable, and the activity of α-amylase was high. With increasing fermentation time, the acidity of the yellow wine rapidly increased, and the activity of α-amylase decreased. After 7 days of fermentation, the α-amylase produced in the fermentation environment fully hydrolyzed the starch to generate reducing sugars, and the saccharification stage was basically completed after 7 days.

**Figure 2:**
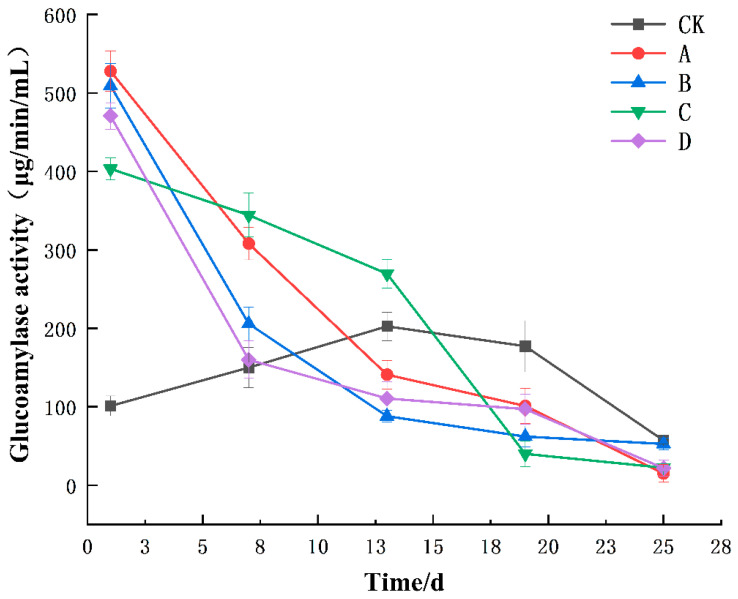
Changes in glucoamylase activity during the fermentation of yellow wine. Groups A, B, C, and D correspond to four starter cultures with different proportions of mixed strains. A (*R. arrhizus* G01:*S. cerevisiae* XDN2:*P. kudriavzevii* XDB1:*L. rhamnosus* SL02 = 4:2:1:1), B (*R. arrhizus* G01:*S. cerevisiae* XDN2:*P. kudriavzevii* XDB1:*L. rhamnosus* SL02 = 5:1:1:1), C (*R. arrhizus* G01:*S. cerevisiae* XDN2:*P. kudriavzevii* XDB1:*L. rhamnosus* SL02 = 4:1:1:2), and D (*R. arrhizus* G01:*S. cerevisiae* XDN2:*P. kudriavzevii* XDB1:*L. rhamnosus* SL02 = 4.5:2.5:0.5:0.5).

Glucoamylase, an α-1,4-glucose hydrolytic amylase, is an enzyme that sequentially hydrolyzes the sugar bond of α-1,4-glucose from the nonreducing end of starch to generate glucose [40,41]. As shown in Figure 2, changes in glucoamylase activity during the yellow wine fermentation were analyzed. The glucoamylase activity in the CK group showed a significant increase (*p* < 0.05) from day 1 to day 13 of fermentation, with a gradual decrease in enzyme activity after day 13. At the beginning of fermentation, the glucoamylase activity of the CK group was significantly lower than that of groups A, B, C, and D (*p* < 0.05). Groups A, B, and D exhibited a general downward trend, experiencing a sharp decline at the onset of fermentation and a gradual decrease towards the conclusion of fermentation. The changes in enzyme activity in Group C gradually decreased during the early and middle stages of fermentation, and the overall enzyme activity was greater than that in the other groups. Glucoamylases are mainly produced by *Rhizopus* [21,42,43]. In the early stages of fermentation, the mold grew and rapidly reproduced and the starch material was rapidly decomposed. However, the activity of glucoamylase gradually decreased during the middle and late stages of fermentation. This may have been caused by a decrease in the mold population as the fermentation progresses [44,45,46]. By the end of fermentation, there were no notable discrepancies in glucoamylase activity across the five groups.

**Figure 3:**
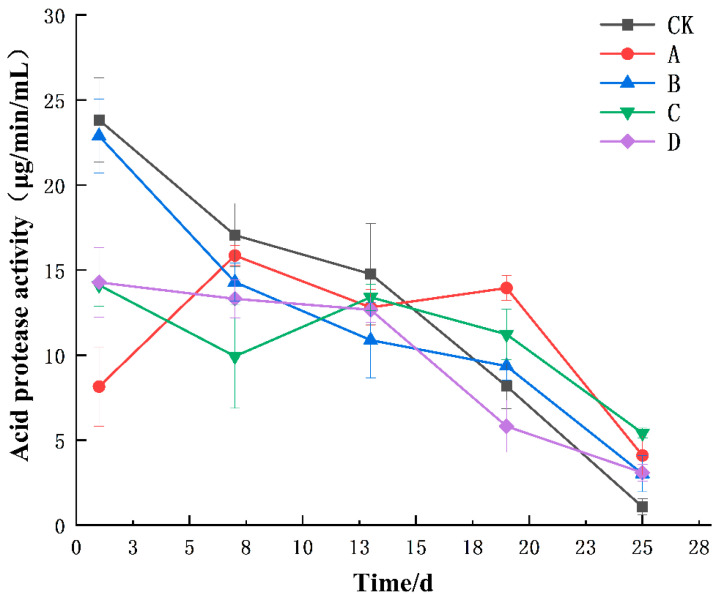
Changes in acid protease activity during the fermentation of yellow wine. Groups A, B, C, and D correspond to four starter cultures with different proportions of mixed strains. A (*R. arrhizus* G01:*S. cerevisiae* XDN2:*P. kudriavzevii* XDB1:*L. rhamnosus* SL02 = 4:2:1:1), B (*R. arrhizus* G01:*S. cerevisiae* XDN2:*P. kudriavzevii* XDB1:*L. rhamnosus* SL02 = 5:1:1:1), C (*R. arrhizus* G01:*S. cerevisiae* XDN2:*P. kudriavzevii* XDB1:*L. rhamnosus* SL02 = 4:1:1:2), and D (*R. arrhizus* G01:*S. cerevisiae* XDN2:*P. kudriavzevii* XDB1:*L. rhamnosus* SL02 = 4.5:2.5:0.5:0.5).

As depicted in Figure 3, the acid protease activities of groups CK, B, and D continuously decreased with the increase in fermentation days. In the pre-fermentation period, the enzyme activity was higher in group CK and the acid protease activity in group A showed a tendency of increasing and then decreasing. At the late stage of fermentation, the enzyme activity of group CK was significantly lower than that of groups A, B, C, and D (*p* < 0.05). Groups A and C still maintained a certain enzyme activity at 25 days of fermentation. This may have been due to the increased production of acid protease in the early stages of fermentation, which aids in the degradation of proteins and other macromolecules, encouraging the development and functioning of microorganisms. Over time, the substrate was gradually consumed, and the activity of the acid protease decreased. The enzyme activity of the remaining groups slowly decreased, which may mean that other microorganisms in the fermentation environment, such as yeast and lactic acid bacteria, gradually dominated in the later stages of fermentation. This lead to a decrease in the number and activity of molds in the fermentation environment. This also reflects the changes in the microbial community structure during the fermentation process.

### 3.2. Physicochemical Properties of Yellow Wine

Reducing sugars, a key substrate of fermentation, were significantly lower in the experimental group than in the commercial yeast mixture group, indicating the effective utilization of sugars (Table 1). Groups A and B differed in microbial proportions, exhibiting significantly lower levels of reducing sugars (3.592 ± 0.2776 g/100 mL and 3.023 ± 0.2002 g/100 mL, respectively), indicating that fermentation efficiency improved. This reduction can be attributed to the synergistic metabolic activities of the microbial symbionts, which enhanced the absorption and conversion of sugars. Total acidity, which is critical for beverage palatability and microbial stability, showed a subtle trend [47,48,49]. Compared with the commercial mixture, the total acidity of the experimental group was slightly lower (5.375 ± 0.187 g/L). This decrease in acidity (6.023 ± 0.668 g/L for Group A, 6.065 ± 0.156 g/L for Group C, and 6.155 ± 0.476 g/L for Group D) may have been affected by the introduction of microorganism-specific metabolic pathways, which could utilize acids in different ways to affect the final acidity profile. Amino acid nitrogen is critical for yeast nutrition and flavor complexity, and it showed a decreasing trend in the experimental group. This trend may indicate that the microbial symbionts assimilated amino acids for protein synthesis and other metabolic functions, which could have affected the trophic dynamics during the fermentation process and ultimately impacted the development of flavor and aroma. The alcohol content was a direct product of sugar fermentation and significantly changed among the different groups. Group B had the highest alcohol concentration (15.1 ± 0.173%vol). This increase could be linked to the adjusted effective fermentation ability of the microbial symbionts, which may have increased ethanol tolerance, allowing for a greater ratio of sugar to ethanol conversion. Sensory evaluation reflects consumers’ views on the overall quality of a product from the perspective of consumers. The experimental group showed an increasing improvement in sensory scores (Group C: 83.125; Group D: 80.313), possibly due to the delicate flavor and aroma developed by the diverse microbial activity. These scores indicate that the microbial symbionts not only affected fermentation kinetics and product composition but also positively contributed to the sensory attributes.

### 3.3. Electronic Tongue Detection

**Figure 4:**
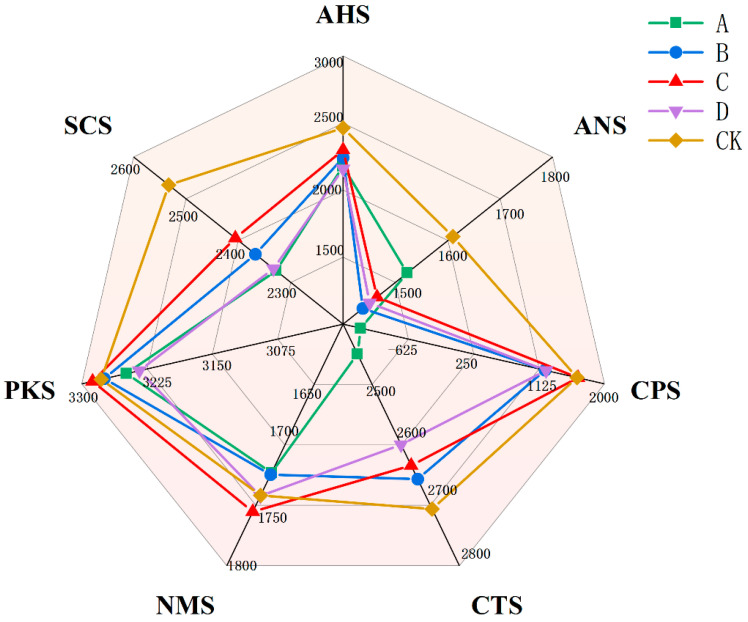
Differences in fermented yellow wines on the e-tongue. Groups A, B, C, and D correspond to four starter cultures with different proportions of mixed strains. A (*R. arrhizus* G01:*S. cerevisiae* XDN2:*P. kudriavzevii* XDB1:*L. rhamnosus* SL02 = 4:2:1:1), B (*R. arrhizus* G01:*S. cerevisiae* XDN2:*P. kudriavzevii* XDB1:*L. rhamnosus* SL02 = 5:1:1:1), C (*R. arrhizus* G01:*S. cerevisiae* XDN2:*P. kudriavzevii* XDB1:*L. rhamnosus* SL02 = 4:1:1:2), and D (*R. arrhizus* G01:*S. cerevisiae* XDN2:*P. kudriavzevii* XDB1:*L. rhamnosus* SL02 = 4.5:2.5:0.5:0.5).

**Figure 5:**
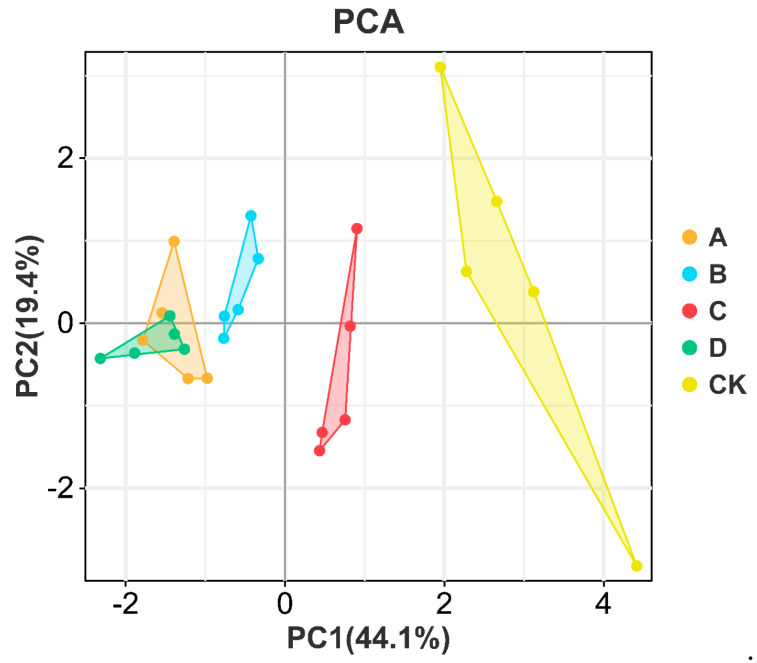
PCA plots of the electronic tongue for different fermented yellow wine samples. Groups A, B, C, and D correspond to four starter cultures with different proportions of mixed strains. A (*R. arrhizus* G01:*S. cerevisiae* XDN2:*P. kudriavzevii* XDB1:*L. rhamnosus* SL02 = 4:2:1:1), B (*R. arrhizus* G01:*S. cerevisiae* XDN2:*P. kudriavzevii* XDB1:*L. rhamnosus* SL02 = 5:1:1:1), C (*R. arrhizus* G01:*S. cerevisiae* XDN2:*P. kudriavzevii* XDB1:*L. rhamnosus* SL02 = 4:1:1:2), and D (*R. arrhizus* G01:*S. cerevisiae* XDN2:*P. kudriavzevii* XDB1:*L. rhamnosus* SL02 = 4.5:2.5:0.5:0.5).

During the fermentation of yellow wine, certain microbial species may promote the production of beneficial flavors while inhibiting the development of undesirable flavors. These subtle differences could be detected by the electronic tongue to evaluate the sensory coordination of yellow wine. As shown in Figure 4, the sour, sweet, salty, and bitter tastes of the commercial yeast mixture were all more intense, and the bitterness was significantly greater than that of the experimental group. The experimental groups had similar flavor profiles, but Group C exhibited a more harmonious sensory characteristics. Commercial mixtures may contain a variety of strains, and the ratio and activity of these strains may not match the flavor characteristics of specific yellow wines. The flavor and taste of yellow wine can be better controlled through the fermentation of carefully blended strains of different origins. To further analyze the differences in taste between groups, the electronic tongue data were subjected to principal component analysis (Figure 5). The data points of Groups A and D were mixed, indicating that their flavors were relatively similar. There were significant differences in taste between the commercial mixture group and the experimental group, and the difference was mainly reflected in the PC1 direction. The differences in taste could be attributed to the different fermentation environments created by different microbial mixing ratios, which produced different types and quantities of flavor compounds, as well as differences in enzymatic hydrolysis products during the later fermentation process.

### 3.4. Volatile Compounds

The aroma of yellow wine is created through the interaction of numerous volatile compounds. Therefore, with different substance types, contents, and ranges, the aroma of yellow wine changes to varying degrees. At present, more than 900 volatile flavor substances, including esters, alcohols, ketones, aldehydes, phenols, and acids, have been detected in Chinese yellow wine [50]. In this study, a total of 77 volatile compounds were detected, among which 37 different volatile compounds were found in the CK group, demonstrating a relatively high diversity of compounds. There were 27 species in Group A and 25 species in Group B. This indicates that under the microbial fermentation system, Rhizopus is the dominant microbe, and when the content is significantly higher than that of the other microbes, the metabolic activity of the remaining microbes may be inhibited to some extent, thus affecting the diversity of the compounds. Thirty-nine compounds were detected in Group C, which had the highest diversity among all groups, while Group D had thirty-three compounds, which also showed high diversity. Based on the calculations (Table 2), the average relative content of the control group (CK) was 2.42%. In contrast to the control group, the experimental groups showed a greater average relative content (A: 3.22%; B: 3.85%; C: 2.49%; D: 2.82%). This may indicate that the synergistic fermentation of multiple strains is an effective method for increasing the content of volatile compounds in yellow wine, mirroring the findings of Yang et al. [51]. The taste and flavor produced by a single strain are not as similar to those of yellow wine fermented with compound strains [52,53].

These compounds are mainly composed of alcohols, esters, acids, ketones, alkanes, and others, with esters being the most abundant. Many research studies have indicated that esters play a beneficial role in enhancing the flavor profile of yellow wine, resulting in a floral and fruity aroma [54]. Specifically, amyl acetate, ethyl caproate, diethyl succinate, phenylethyl acetate, and ethyl palmitate are all common esters found in yellow wine. They can contribute different aroma characteristics, such as a honey-like aroma and a rose aroma, which give yellow wine its unique flavors. These volatile compounds were all detected in the studied yellow wine, in line with the findings of prior research [55]. Among all the groups, hexadecanoic acid ethyl ester was the volatile compound with the highest content. This indicates that this compound was the dominant compound under different treatment conditions and may have had the most significant effect on the flavor of the yellow wine sample.

In addition, some esters not found in commercial yeast blends, such as ethyl hexanoate, ethyl heptadecanoate, 9-hexadecenoic acid ethyl ester, ethyl linoleate, and pentyl decanoate, were detected in the yellow wines fermented by mixed strains with a set ratio. The specific expression of these esters in yellow wine is affected by other flavor components in yellow wine and their changes during different fermentation stages. This may indicate that this set of treatment conditions can promote the formation of certain esters. There were more types of esters, possibly because the esters in yellow wine are formed by dehydration and condensation under the interaction of alcohols and acids or because the fermentation raw materials are rich in starch, which is decomposed under the action of glucoamylases into glucose. Glucose then generates pyruvate via glycolysis, which is then oxidatively decarboxylated to generate acetyl-CoA. Acetyl-CoA then synthesizes esters with corresponding alcohols under the action of alcohol acyltransferase, thereby increasing the diversity of esters in yellow wine [56,57,58].

Alcohols typically contribute more to the aroma of wine, particularly phenylethanol, which is renowned for its rose scent [59]. Phenyl alcohol is mainly synthesized through the shikimate pathway, the Ehrlich pathway, or the phenylethylamine pathway [60]. The first two pathways both generate phenylpyruvate, which is then converted to phenylethanol through phenylacetaldehyde, while the phenylethylamine pathway can directly synthesize phenylacetaldehyde. Phenylpyruvate decarboxylase (EC:4.1.1.-), which catalyzes the decarboxylation of phenylpyruvate to phenylacetaldehyde, is from the genera *Pichia*, *Meyerozyma*, and *Clavispora*. This decarboxylase plays a crucial role in the Ehrlich pathway. The Ehrlich pathway is the preferred pathway for the synthesis of phenylethanol by microorganisms and is widely found in various yeasts [61]. In this study, the relative content of phenylethanol in the CK group was 29.71%, which was significantly higher than what was observed in the experimental groups (A: 14.76%, B: 14.2%, C: 13.12%, D: 16.29%). The possible reason for this phenomenon is that *Saccharomyces cerevisiae* or some types of yeast produce a certain amount of phenylpyruvate decarboxylase, resulting in increased phenylethanol production [62,63,64].

A small amount of compounds, such as acids and ketones, were also detected in the yellow wine samples. Acetic acid is the main volatile acid in yellow wine and provides a sense of stimulation. Non-volatile acids, such as lactic acid, succinic acid, and citric acid, can give the wine a mellow sense and aftertaste. During the aging process of yellow wine, some acids may be decomposed by microorganisms or react with other components, resulting in a reduction in the types and contents of acids. Yellow wine contains small amounts of ketones, but they also contribute to its flavor. Ketones usually endow yellow wine with a fruity or floral aroma and increase the complexity of its flavor. The roles of alkane compounds in yellow wine are relatively insignificant, and they usually do not provide significant flavor characteristics. However, some alkanes may have certain effects on the stability and preservation of wine [65].

### 3.5. Amino Acid Analysis

Yellow wine contains a large amount of amino acids, which is why it is called a “liquid cake” [66,67]. During the wine-making process, microorganisms use proteases to enzymatically hydrolyze proteins in wine-making raw materials, generating amino acids. This phenomenon can also be observed during the autolysis process of yeast, as well as in the physiological activities of different microorganisms [68]. Yellow wine’s flavor is greatly influenced by amino acids, which offer a variety of tastes including sweet, bitter, umami, sour, and salty. Additionally, these amino acids contribute to the wine’s appealing color. This is due to amino acids providing a source of nitrogen for biochemical reactions [69]. Therefore, changes in amino acids in yellow wine may have a certain impact on the product’s quality. According to Zhao, Rotzoll et al. [70,71], the taste of amino acids can be categorized into four groups: umami, sweet, bitter, and salty amino acids. Umami amino acids include aspartic acid (Asp) and glutamate (Glu). Sweet amino acids include alanine (Ala), glycine (Gly), serine (Ser), and threonine (Thr). Bitter amino acids include histidine (His), lysine (Lys), valine (Val), tyrosine (Tyr), leucine (Leu), isoleucine (Ile), phenylalanine (Phe), and arginine (Arg). Salty amino acids include cysteine (Cys) and methionine (Met).

The yellow wines brewed in this study all contained 17 kinds of amino acids, as shown in Table 3. The contents of Asp, Glu, Ala, Leu, Arg, and Pro were all high, which is consistent with the study of Zhang et al. [72]. The difference was that the average content of these six amino acids was greater than 1.0 mg/mL, which differs from the results reported in the literature [67,68]. This difference may have been caused by variations in the used promoter strains and raw materials. Our findings revealed that the free amino acid levels in yellow wine were significantly higher compared with those in other types of wine. Additionally, the free amino acid content in the yellow wine fermented using a controlled strain ratio was also significantly higher than that of conventionally fermented yellow wine [69,73]. Interestingly, we found that the levels of bitter amino acids (Phe, Ile, Leu, and Lys) in the co-fermented yellow wine were slightly lower than those in the control group when using *R. arrhizus*, *S. cerevisiae*, *P. kudriavzevii*, and *L. rhamnosus*. The Ehrlich pathway is a way of amino acid catabolism in which amino acids are first converted to α-keto acids by transamination and then to the corresponding heteroaldehydes by decarboxylation. Finally, the heteroaldehyde can be reduced to the corresponding alcohol or oxidized under certain conditions. Phenylethanol, 2-methylbutanol, and 3-methylbutanol are all derived from the corresponding amino acids via the Ehrlich pathway. A decrease in the amount of Phe, a key substrate in the Ehrlich pathway, directly reduces the production of α-keto acids, which may lead to a corresponding change in the yield of volatile compounds [74,75,76].

**Figure 6:**
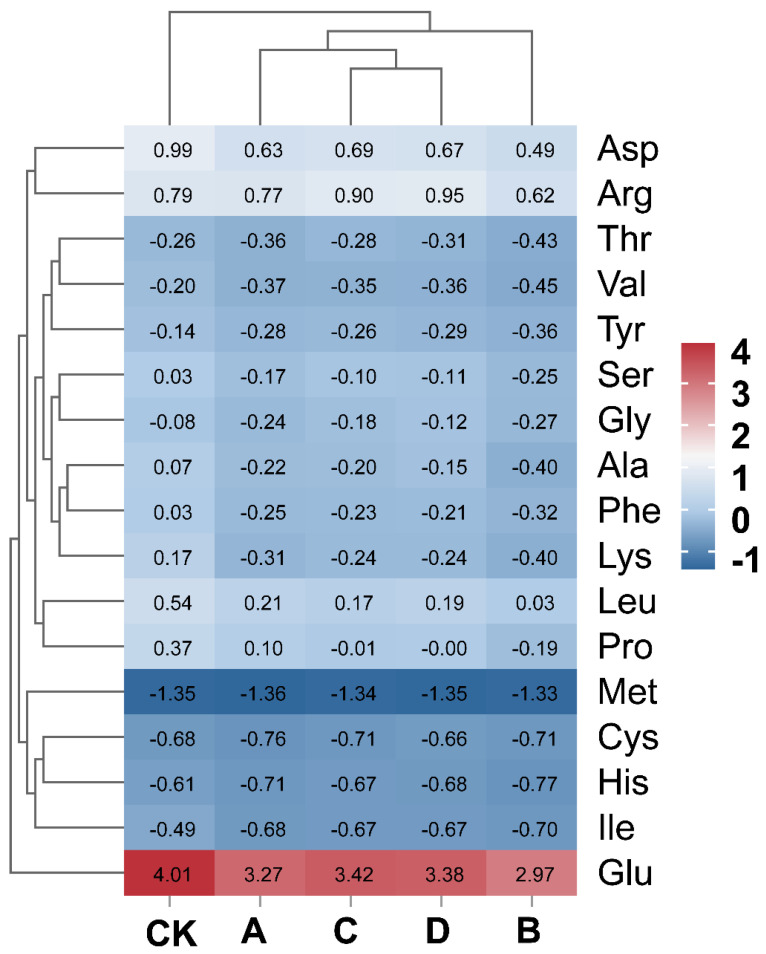
Heatmap of amino acid content in yellow wine fermented with different composite strain ratios. Red: relatively high amino acid content; blue: relatively low amino acid content. Groups A, B, C, and D correspond to four starter cultures with different proportions of mixed strains. A (*R. arrhizus* G01:*S. cerevisiae* XDN2:*P. kudriavzevii* XDB1:*L. rhamnosus* SL02 = 4:2:1:1), B (*R. arrhizus* G01:*S. cerevisiae* XDN2:*P. kudriavzevii* XDB1:*L. rhamnosus* SL02 = 5:1:1:1), C (*R. arrhizus* G01:*S. cerevisiae* XDN2:*P. kudriavzevii* XDB1:*L. rhamnosus* SL02 = 4:1:1:2), and D (*R. arrhizus* G01:*S. cerevisiae* XDN2:*P. kudriavzevii* XDB1:*L. rhamnosus* SL02 = 4.5:2.5:0.5:0.5).

There are eight essential amino acids (Leu, Val, Ser, Thr, Met, Phe, Ile, and Lys) in yellow wine, of which the CK group accounted for 36.5% of the total amount, Group A accounted for 35.1%, Group B accounted for 35.4%, Group C accounted for 35.2%, and Group D accounted for 35.0%. A heatmap was constructed based on the normalized data as a basis for visualizing the distribution of amino acids in different yellow wines (Figure 6). Each small square represents an amino acid in the sample, and its color change represents the content of that amino acid. In all samples, it was evident that the Glu content was significantly higher compared with the other amino acids. During the brewing process, the proteins in the raw material are hydrolyzed to peptides and amino acids by the action of proteases produced by yeasts and bacteria. Among them, glutamate is one of these hydrolysis products. These amino acids are subsequently further converted into other biologically active substances through the action of various enzymes in the metabolic process of microorganisms. This is closely connected to the creation of certain flavor compounds. For example, glutamate can generate ammonia under the action of glutamate dehydrogenase, which can react with 2,3-butanediol to produce the flavor substance tetramethylpyrazine [77].

## 4. Conclusions

Based on the study findings, incorporating *R. arrhizus*, *S. cerevisiae*, *P. kudriavzevii*, and *L. rhamnosus* in mixed fermentation can significantly improve the taste and overall quality of highland barley yellow wine. At a controlled bacterial load of 1 × 10^6^ CFU/mL, adjusting the ratio of *R. arrhizus*:*S. cerevisiae*:*P. kudriavzevii*:*L. rhamnosus* to 4:1:1:2 can effectively improve the physicochemical characteristics and sensory attributes of yellow wine, resulting in a balanced sweet and sour taste. The percentage of reduced sugar was moderate (reduced sugar: 3.977 ± 0.0544 g/100 mL; total acid: 6.065 ± 0.156 g/L; alcohol content: 13.1 ± 0.153%vol). Through the compounding of strains and the adjustment of the strain ratio, enzyme activity was maintained at a certain level in the later stage of yellow wine fermentation to sustain the secondary fermentation of yellow wine. In addition, multistrain synergistic fermentation can effectively increase the content of volatile compounds in yellow wine, with hexadecanoic acid ethyl ester as the main component. This indicates that these substances may have the most significant impact on the flavor of yellow wine under different treatment conditions. Additionally, all four studied highland barley yellow wines contained 17 common amino acids, with glutamic acid being the most abundant. Future research can further investigate the effects of different microbial ratios on the fermentation process and quality of yellow wine, especially the mechanism of microbial interaction.

## Figures and Tables

**Table 1:** Comparison of physicochemical parameters between composite-strain-fermented yellow wine and ordinary fermented yellow wine.

Clusters	Mixed Bacteria Ratio (*R. arrhizus*:*S. cerevisiae*:*P. kudriavzevii*:*L. rhamnosus*)				Reduced Sugar (g/100 mL)	Total Acid (g/L)	Amino Acid Nitrogen (g/L)	Alcohol Content (%vol)	Sense Value
CK	Commercially available koji				6.503 ± 0.6590 a	7.175 ± 0.137 a	0.658 ± 0.014 a	10.5 ± 0.500 d	78.5
A	4	2	1	1	3.592 ± 0.2776 bc	6.023 ± 0.668 bc	0.294 ± 0.024 cd	12.0 ± 0.252 c	79.6
B	5	1	1	1	3.023 ± 0.2002 c	5.375 ± 0.187 c	0.266 ± 0.014 d	15.1 ± 0.173 a	80.1
C	4	1	1	2	3.977 ± 0.0544 b	6.065 ± 0.156 bc	0.313 ± 0.021 c	13.1 ± 0.153 b	83.1
D	4.5	2.5	1	0.5	3.889 ± 0.3446 b	6.155 ± 0.476 b	0.331 ± 0.035 b	13.4 ± 0.416 b	80.3

Results represent the mean ± SD for three independent experiments. Values in the same row with different letters are significantly different (*p* < 0.05). Groups A, B, C, and D correspond to four starter cultures with different proportions of mixed strains. A (*R. arrhizus* G01:*S. cerevisiae* XDN2:*P. kudriavzevii* XDB1:*L. rhamnosus* SL02 = 4:2:1:1), B (*R. arrhizus* G01:*S. cerevisiae* XDN2:*P. kudriavzevii* XDB1:*L. rhamnosus* SL02 = 5:1:1:1), C (*R. arrhizus* G01:*S. cerevisiae* XDN2:*P. kudriavzevii* XDB1:*L. rhamnosus* SL02 = 4:1:1:2), and D (*R. arrhizus* G01:*S. cerevisiae* XDN2:*P. kudriavzevii* XDB1:*L. rhamnosus* SL02 = 4.5:2.5:0.5:0.5).

**Table 2:** Relative contents of volatile compounds in fermented yellow wines with different ratios of compounding strains (presumptive).

	Volatile Compound	CAS	Chemical Formula	Relative Content/%				
				CK	A	B	C	D
Alcohol compounds (6)	1-Propanol, 2,2-dimethyl-	75-84-3	C_5_H_12_O	0.34	/	/	/	/
	Phenylethyl Alcohol	60-12-8	C_8_H_10_O	29.71	14.76	14.2	13.12	16.29
	2-Naphthalenemethanol, 2,3,4,4a,5,6,7,8-octahydro-.alpha.,.alpha.,4a,8-tetramethyl-, [2R-(2.alpha.,4a.beta.,8.beta.)]-	63891-61-2	C_15_H_26_O	/	0.08	/	/	/
	1,1,3,3,5,5,7,7,9,9-Decamethyl-9-(2-methylpropoxy)pentasiloxan-1-ol	ND	C_14_H_40_O_6_Si_5_	/	/	/	0.03	/
	1,1,3,3,5,5,7,7-Octamethyl-7-(2-methylpropoxy)tetrasiloxan-1-ol		C_12_H_34_O_5_Si_4_	/	0.02	/	/	/
	1-Heptanol, 2,4-dimethyl-,	98982-97-9	C_9_H_20_O	2.77	1.7	/	1.11	/
Ester compounds (37)	1-Butanol, 3-methyl-, propanoate	105-68-0	C_8_H_16_O_2_	/	/	/	0.27	/
	1-Butanol, 2-methyl-, propanoate	2438-20-2	C_8_H_16_O_2_	/	/	/	0.1	/
	Acetic acid, pentyl ester	628-63-7	C_7_H_14_O_2_	0.57	1.19	0.67	0.92	0.49
	Hexanoic acid, ethyl ester	123-66-0	C_8_H_16_O_2_	/	0.3	/	0.13	0.08
	2-Propenoic acid, 2-methyl-, pentyl ester	2849-98-1	C_9_H_16_O_2_	0.1	/	/	/	/
	Formic acid, octyl ester	112-32-3	C_9_H_18_O_2_	0.07	/	/	/	/
	Butanedioic acid, diethyl ester	123-25-1	C_8_H_14_O_4_	0.2	1.02	0.8	0.61	0.81
	Octanoic acid, ethyl ester	106-32-1	C_10_H_20_O_2_	0.41	0.82	0.47	0.57	0.45
	Acetic acid, 2-phenylethyl ester	103-45-7	C_10_H_12_O_2_	1.72	5.08	5.84	4.33	6.35
	Nonanoic acid, ethyl ester	123-29-5	C_11_H_22_O_2_	0.23	0.15	/	0.09	/
	Decanoic acid, ethyl ester	110-38-3	C_12_H_24_O_2_	0.23	1.33	1.03	1.76	0.97
	3,4-Dihydroxymandelic acid, ethyl ester, tri-TMS	ND	C_19_H_36_O_5_Si_3_	0.27	0.47	2.11	0.27	1.47
	Methyl 2-methyl-2-(methoxy-3-hydroxypropoxy)amino-propanoate	76664-32-9	C_9_H_19_NO_5_	0.05	/	/	/	/
	Dodecanoic acid, ethyl ester	106-33-2	C_14_H_28_O_2_	0.44	/	0.91	1.51	1.07
	Tetradecanoic acid, ethyl ester	124-06-1	C_16_H_32_O_2_	2.62	2.38	2.67	3.51	3.23
	Formic acid, undecyl ester		C_12_H_24_O_2_	0.11	/	/	/	/
	Pentadecanoic acid, ethyl ester	41114-00-5	C_17_H_34_O_2_	0.15	0.12	/	/	/
	Ethyl 9-hexadecenoate	54546-22-4	C_18_H_34_O_2_	/	0.46	0.42	0.56	0.59
	Hexadecanoic acid, ethyl ester	628-97-7	C_18_H_36_O_2_	31.65	43.22	41.86	42.51	34.06
	i-Propyl 14-methyl-pentadecanoate	ND	C_19_H_38_O_2_	0.37	0.13	0.09	/	/
	Hexadecanoic acid, 2-methylpropyl ester	110-34-9	C_20_H_40_O_2_	0.08	0.11	0.12	0.2	/
	Heptadecanoic acid, ethyl ester	14010-23-2	C_19_H_38_O_2_	/	0.09	0.75	0.12	0.05
	Butyl 9,12-octadecadienoate	ND	C_22_H_40_O_2_	3.8	/	/	/	/
	Ethyl Oleate	111-62-6	C_20_H_38_O_2_	3.8	12.67	12.26	9.19	9.99
	9,12-Octadecadienoic acid, ethyl ester	7619-08-1	C_20_H_36_O_2_	/	/	0.5	/	0.58
	(Z)-Ethyl pentadec-9-enoate	56219-09-1	C_17_H_32_O_2_	/	/	0.1	0.2	0.14
	Ethyl 13-methyl-tetradecanoate	64317-63-1	C_17_H_34_O_2_	/	/	0.13	/	0.14
	trans,trans-9,12-Octadecadienoic acid, propyl ester	ND	C_21_H_38_O_2_	/	/	9.61	/	/
	O-Butylisourea	57536-14-8	C_5_H_12_N_2_O	/	/	/	1.91	/
	Arsenous acid, tris(trimethylsilyl) ester	55429-29-3	C_9_H_27_AsO_3_Si_3_	/	/	/	0.02	/
	Octadecanoic acid, ethyl ester	111-61-5	C_20_H_40_O_2_	/	/	/	0.12	/
	Pentadecanoic acid, 3-methylbutyl ester	2306-91-4	C_15_H_30_O_2_	/	/	/	0.1	/
	1-Undecanol, acetate	1731-81-3	C_13_H_26_O_2_	/	/	/	0.06	/
	l-(+)-Ascorbic acid 2,6-dihexadecanoate	28474-90-0	C_38_H_68_O_8_	2.73	/	/	1.97	/
	Heptadecanoic acid, 15-methyl-, ethyl ester	57274-46-1	C_20_H_40_O_2_	/	/	/	/	1.74
	Decanoic acid, pentyl ester	5933-87-9	C_15_H_30_O_2_	/	/	/	/	0.13
	cis-10-Pentadecenoic acid, propyl ester	ND	C_18_H_34_O_2_	/	/	/	/	0.08
Acid compounds (5)	DL-Allothreonine	144-98-9	C_4_H_9_NO_3_	3.8	/	/	/	/
	Butanoic acid, 4-butoxy-	55724-73-7	C_8_H_16_O_3_	0.02	/	/	/	/
	Tetradecanoic acid	544-63-8	C_14_H_28_O_2_	0.24	/	/	0.05	0.09
	11-Bromoundecanoic acid	2834-05-1	C_11_H_21_BrO_2_	/	0.07	/	/	/
	2,5-Dihydroxybenzoic acid, 3TMS derivative	3618-20-0	C_16_H_30_O_4_Si_3_	/	/	0.8	0.07	1.16
Alkane compounds (10)	Dodecane, 2,6,11-trimethyl-	31295-56-4	C_15_H_32_	/	/	/	/	0.12
	Heptane, 3-ethyl-5-methylene-	52896-90-9	C_10_H_20_	0.07	/	/	/	/
	Decane, 2,3,6-trimethyl-	62238-12-4	C_13_H_28_	0.16	/	/	/	/
	Eicosane	112-95-8	C_20_H_42_	0.32	0.18	/	/	/
	Cyclopentane, (4-octyldodecyl)-	5638-09-5	C_25_H_50_	0.1	/	/	/	/
	2,6,10-Trimethyltridecane	3891-99-4	C_16_H_34_	0.2	/	/	/	/
	Decane, 2,3,7-trimethyl-	62238-13-5	C_13_H_28_	/	0.13	0.05	/	/
	3,6-Dioxa-2,4,5,7-tetrasilaoctane, 2,2,4,4,5,5,7,7-octamethyl-	ND	C_10_H_30_O_2_Si_4_	/	0.16	0.07	/	0.14
	Cyclohexane, 1,2-dimethyl-3-pentyl-4-propyl-	62376-17-4	C_16_H_32_	/	0.03	/	/	/
	2-Methyltetracosane	1560-78-7	C_25_H_52_	/	/	0.38	0.42	/
Ketones (3)	3-Octanone	106-68-3	C_8_H_16_O	0.1	/	/	/	/
	2H-Benzocyclohepten-2-one, decahydro-9a-methyl-, trans-	55103-67-8	C_12_H_20_O	0.61	/	/	/	/
	Neronine, 4.beta.,5-dihydro-	19483-30-8	C_18_H_21_NO_6_	/	0.08	/	/	/
Other compounds (16)	Oxime-, methoxy-phenyl-_	ND	C_8_H_9_NO_2_	0.97	/	/	0.1	0.14
	2′,6′-Dihydroxyacetophenone, acetate	ND	C_10_H_10_O_4_	0.14	/	/	/	/
	i-Propyl tricosanoate	ND	C_26_H_52_O_2_	0.22	/	/	/	/
	Benzeneethanamine, N-[(pentafluorophenyl)methylene]-.beta.,3,4-tris[(trimethylsilyl)oxy]-	55429-13-5	C_24_H_34_F_5_NO_3_Si_3_	0.2	0.06	/	0.04	0.04
	2H-3,9a-Methano-1-benzoxepin, octahydro-2,2,5a,9-tetramethyl-, [3R-(3.alpha.,5a.alpha.,9.alpha.,9a.alpha.)]-	5956-09-2	C_15_H_26_O	/	/	0.03	/	0.05
	1,2-Ethanediamine, N-(phenylmethyl)-	4152-09-4	C_9_H_14_N_2_	/	/	/	0.09	/
	(Z,Z)-.alpha.-Farnesene	ND	C_15_H_24_	/	/	/	0.22	/
	6-epi-shyobunol	69350-61-4	C_15_H_26_O	/	/	0.43	0.78	/
	Ethyl 9.cis.,11.trans.-octadecadienoate	ND	C_20_H_36_O_2_	/	/	/	0.87	1.32
	3-N-Nitroso-solanocapsine	ND	C_27_H_45_N_3_O_3_	/	/	/	0.18	/
	Citronellol epoxide (R or S)	ND	C_10_H_20_O_2_	/	/	/	0.11	/
	n-Propyl 9,12-octadecadienoate	ND	C_21_H_38_O_2_	/	/	/	8.86	9.93
	Thiophene, tetrahydro-2-methyl-	1795-09-1	C_5_H_10_S	/	/	/	/	0.13
	.tau.-Muurolol	19912-62-0	C_15_H_26_O	/	/	/	/	0.2
	2(1H)-Naphthalenone, octahydro-4a,5-dimethyl-, (4a.alpha.,5.alpha.,8a.beta.)-	51557-64-3	C_12_H_20_O	/	/	/	/	0.91
	Gamolenic acid	506-26-3	C_18_H_30_O_2_	/	/	/	/	0.14

Groups A, B, C, and D correspond to four starter cultures with different proportions of mixed strains. A (*R. arrhizus* G01:*S. cerevisiae* XDN2:*P. kudriavzevii* XDB1:*L. rhamnosus* SL02 = 4:2:1:1), B (*R. arrhizus* G01:*S. cerevisiae* XDN2:*P. kudriavzevii* XDB1:*L. rhamnosus* SL02 = 5:1:1:1), C (*R. arrhizus* G01:*S. cerevisiae* XDN2:*P. kudriavzevii* XDB1:*L. rhamnosus* SL02 = 4:1:1:2), and D (*R. arrhizus* G01:*S. cerevisiae* XDN2:*P. kudriavzevii* XDB1:*L. rhamnosus* SL02 = 4.5:2.5:0.5:0.5). ND: Not Found/Not Detected.

**Table 3:** Amino acid content of yellow wine fermented with different composite strain ratios.

Amino Acids	CK	A	B	C	D
			(mg/mL)		
Asp	1.383 ± 0.033 a	1.18 ± 0.057 bc	1.103 ± 0.062 c	1.215 ± 0.050 b	1.201 ± 0.041 bc
Thr	0.686 ± 0.014 a	0.628 ± 0.027 bc	0.593 ± 0.012 c	0.675 ± 0.023 a	0.657 ± 0.018 ab
Ser	0.845 ± 0.025 a	0.737 ± 0.037 bc	0.692 ± 0.023 c	0.776 ± 0.035 b	0.772 ± 0.028 b
Glu	3.061 ± 0.079 a	2.647 ± 0.117 bc	2.48 ± 0.068 c	2.734 ± 0.097 b	2.711 ± 0.068 b
Gly	0.787 ± 0.026 a	0.699 ± 0.043 c	0.681 ± 0.028 c	0.733 ± 0.031 bc	0.764 ± 0.022 a
Ala	0.872 ± 0.027 a	0.709 ± 0.046 b	0.61 ± 0.025 c	0.717 ± 0.043 b	0.75 ± 0.024 b
Cys	0.453 ± 0.019 a	0.407 ± 0.013 b	0.435 ± 0.014 ab	0.436 ± 0.025 ab	0.466 ± 0.016 a
Val	0.721 ± 0.031 a	0.627 ± 0.023 b	0.578 ± 0.012 b	0.634 ± 0.034 b	0.63 ± 0.035 b
Met	0.083 ± 0.016 a	0.072 ± 0.015 a	0.091 ± 0.009 a	0.087 ± 0.008 a	0.081 ± 0.007 a
Ile	0.558 ± 0.015 a	0.452 ± 0.023 b	0.44 ± 0.012 b	0.457 ± 0.035 b	0.456 ± 0.011 b
Leu	1.132 ± 0.044 a	0.948 ± 0.037 b	0.85 ± 0.022 c	0.923 ± 0.048 bc	0.937 ± 0.030 b
Tyr	0.754 ± 0.013 a	0.673 ± 0.018 bc	0.631 ± 0.012 c	0.687 ± 0.036 b	0.67 ± 0.025 bc
Phe	0.85 ± 0.024 a	0.694 ± 0.022 bc	0.654 ± 0.036 c	0.705 ± 0.021 b	0.712 ± 0.019 b
His	0.493 ± 0.017 a	0.436 ± 0.013 bc	0.405 ± 0.012 c	0.456 ± 0.026 b	0.452 ± 0.018 b
Lys	0.927 ± 0.012 a	0.66 ± 0.023 b	0.611 ± 0.026 c	0.696 ± 0.035 b	0.697 ± 0.021 b
Arg	1.271 ± 0.033 ab	1.26 ± 0.053 ab	1.174 ± 0.069 b	1.329 ± 0.054 a	1.356 ± 0.022 a
Pro	1.034 ± 0.048 a	0.888 ± 0.037 b	0.724 ± 0.024 c	0.826 ± 0.043 b	0.828 ± 0.035 b
total	15.91 ± 0.641 a	13.717 ± 0.364 bc	12.752 ± 0.298 c	14.086 ± 0.357 b	14.14 ± 0.338 b

Groups A, B, C, and D correspond to four starter cultures with different proportions of mixed strains. A (*R. arrhizus* G01:*S. cerevisiae* XDN2:*P. kudriavzevii* XDB1:*L. rhamnosus* SL02 = 4:2:1:1), B (*R. arrhizus* G01:*S. cerevisiae* XDN2:*P. kudriavzevii* XDB1:*L. rhamnosus* SL02 = 5:1:1:1), C (*R. arrhizus* G01:*S. cerevisiae* XDN2:*P. kudriavzevii* XDB1:*L. rhamnosus* SL02 = 4:1:1:2), and D (*R. arrhizus* G01:*S. cerevisiae* XDN2:*P. kudriavzevii* XDB1:*L. rhamnosus* SL02 = 4.5:2.5:0.5:0.5). Results represent the mean ± SD for three independent experiments. Values in the same row with different letters are significantly different (*p* < 0.05).

## Data Availability

The original contributions presented in the study are included in the article, further inquiries can be directed to the corresponding authors.
